# Comparative performance of reference laboratory tests and in-clinic tests for *Giardia* in canine feces

**DOI:** 10.1186/s13071-018-2990-6

**Published:** 2018-08-01

**Authors:** Jennifer Mizhquiri Barbecho, Dwight D. Bowman, Janice L. Liotta

**Affiliations:** College of Veterinary Medicine, Cornell University, C4-114 VMC, 930 Campus Rd, Ithaca, NY 14853 USA

**Keywords:** *Giardia*, *Giardia* diagnostics, Fecal assay, Antigen assay, In-clinic diagnostics, Point of care diagnostics, Canine disease

## Abstract

**Background:**

We examined the performance of four in-clinic *Giardia* diagnostic tests by comparing results to three laboratory methods for detection of *Giardia*. A set of 177 fecal samples originally submitted to a commercial laboratory by veterinarians for routine ova and parasite (O&P) testing was used. Specimens were examined by direct immunofluorescence assay (DFA) for presence of *Giardia* cysts which served as the gold standard. Fecal samples were tested using a *Giardia*-specific cyst wall antigen microtiter plate format enzyme-linked immunosorbent assay (ELISA) and each of the in-clinic assays adhering to the package insert for each kit.

**Results:**

Evaluated were four in-clinic antigen test kits: VetScan® Canine *Giardia* Rapid Test (Abaxis), Anigen® Rapid CPV-CCV-*Giardia* Antigen Test (BioNote), SNAP® *Giardia* Test (IDEXX) and Witness® *Giardia* Test (Zoetis). In the comparison of the in-clinic tests to the DFA standard test sensitivity ranged between 70.0–87.1%, and specificity ranged between 71.1–93.4%.

**Conclusion:**

Of the tests evaluated here, the SNAP test had the highest sensitivity and specificity. The SNAP test had the highest percent positive and percent negative agreement when compared to the microtiter plate format ELISA and the O&P assay.

## Background

*Giardia* are protozoan parasites capable of infecting numerous mammals including both dogs and cats and has been associated with gastrointestinal disease [1–3]. For dogs, diarrhea is a common clinical manifestation presented to veterinary clinics and a consideration in managing these patients is determining *Giardia* infection status. *Giardia* can be identified in feces by visualization of whole cysts by microscopy using zinc sulfate (ZnSO_4_) fecal floatation [ova and parasite (O&P) test], by direct immunofluorescence (DFA) using *Giardia*-specific fluorescent antibodies or by demonstrating the presence of *Giardia*-specific antigen in feces using an immunoassay [4–6].

Fecal flotation can be performed in-clinic but identification of cysts can be difficult because of yeast and debris in the fecal sample which resemble cysts and because shedding is intermittent requiring multiday examinations [7]. Direct immunofluorescence assay has been shown to be more sensitive and specific than conventional flotation methods and herein served as the gold standard method for identifying fecal *Giardia* [5, 8, 9]. The DFA requires a fluorescent microscope limiting its usefulness in-clinic.

*Giardia* antigen detection tests detect a secreted soluble *Giardia*-specific cyst wall antigen and are available from several sources [3, 6, 7]. These tests are available as microtiter plate format ELISAs which can be read visually or by an instrument-based procedure in a reference laboratory setting and single use rapid tests which are read visually and are used in-clinic. A number of in-clinic tests have been described and are now available for use but studies directly comparing the performance of these commercially available tests have not been reported [6, 10, 11]. Using DFA as the gold standard, we examined the performance of four in-clinic rapid tests using a set of clinic samples originally submitted to a commercial laboratory by veterinarians for conventional ova and parasite (O&P) testing. Evaluated were four in-clinic antigen test kits: VetScan® Canine *Giardia* Rapid Test (Abaxis), Anigen® Rapid CPV-CCV-*Giardia* Antigen Test (BioNote), SNAP® *Giardia* Test (IDEXX) and Witness® *Giardia* Test (Zoetis).

## Methods

### Canine samples

Fecal samples were sourced from IDEXX Reference Laboratories (IRL) by the sole criterion that they were submitted by practicing veterinarians for general ova and parasite (O&P) testing which was performed at the reference laboratory using ZnSO_4_ centrifugal floatation. No clinical information was available. Samples were stored frozen (-20 °C) after the initial O&P test. A total of 87 O&P positive and 90 O&P negative samples was randomly selected so approximately an equal number of O&P positive and negative samples were obtained. Technicians performing all remaining testing had no knowledge of the O&P results.

### In-clinic tests

Evaluated were four in-clinic antigen rapid test kits: VetScan® Canine *Giardia* Rapid Test (Abaxis, Union City, USA), Anigen® Rapid CPV-CCV-*Giardia* Antigen Test (BioNote, Seoul, Korea), SNAP® *Giardia* Test (IDEXX, Westbrook, USA) and Witness® *Giardia* Test (Zoetis, Parsippany, USA). The four kits were tested concurrently adhering to the package insert for each kit. Before testing samples were randomized and blind-labeled. There were no additional freeze-thaw cycles or sample handling differences in testing events between the four rapid tests. Visual interpretation of results was performed following the manufacturers’ instructions supplied with each test kit.

### Microtiter plate format ELISA

Samples were tested with the ProSpecT® *Giardia*/*Cryptosporidium* Microplate Assay (Thermo Fisher Scientific, Pittsburgh, USA) using instructions supplied with the kit. Plates were read spectrophotometrically at 450 nm; samples were considered positive when the net optical density reading (sample OD-negative control OD) was ≥ 0.05.

### DFA test

The DFA was conducted and *Giardia* cysts were counted using a previously described procedure with modifications [5]. Briefly, 0.1 g feces was added to 900 μl 0.02 M sodium phosphate-buffered saline pH 7.4 (PBS) and mixed thoroughly. An aliquot of the mixed sample (100 μl) was added to a second tube containing 900 μl of PBS. The second tube was thoroughly mixed and 100 μl was added to a third tube which was mixed with 5 μl of Merifluor® detection reagent (Meridian Biosciences, Cincinnati, USA). The sample was then incubated at room temperature for 30 min in the dark and then maintained at 4 °C until the quantitative cyst count was performed. A sample volume of 10.5 μl was removed and examined using a fluorescent microscope. The cysts were counted and a total cysts/gram was calculated. A positive and negative control were read prior to each test. The positive control was 85 μl PBS, 15 μl positive control from the Merifluor kit, and 5 μl Merifluor detection reagent. The negative control consisted of 100 μl PBS and 5 μl Merifluor detection reagent.

### Data collection and analysis

Statistical analysis was performed in SAS© version 9.4 (SAS Institute, Inc., Cary, NC, USA). Agresti-Coull 95% confidence intervals were used for calculation of sensitivity and specificity compared to the DFA reference method, as well as percent positive and negative agreement to the microtiter plate format ELISA test. Agresti-Coull confidence intervals were calculated using the FREQ procedure in SAS 9.4. Prevalence adjusted test agreement was calculated using the following formula: agreement = prevalence × sensitivity + (1 – prevalence) × specificity. In this calculation, we used the prevalence (5.4%) found for client-owned symptomatic dogs presented to a veterinary hospital as determined by a monoclonal antibody-based immunofluorescent antibody assay [12].

## Results

### DFA, O&P and microtiter plate ELISA results

The final sample set consisted of 177 samples; all were examined by DFA and by microtiter plate format ELISA. Cysts were identified by DFA in 101 of 177 samples which were classified as positive and were not identified in 76 samples which were classified as negative. DFA results were compared to the original O&P results reported by the reference lab. In the O&P test, cysts were identified in 82 of the 101 DFA positive samples (sensitivity 81.2%) and in 5 of the 76 DFA negative samples (specificity 93.4%). In the microtiter plate format ELISA, 95 of the 101 DFA reactive samples were positive (sensitivity 94.1%) and 74 of the 76 DFA negative samples were negative (specificity 97.4%). A comparison of results for the O&P and microtiter plate test are shown in Table 1.

**Table 1:** Statistical comparison of ova and parasite results and reference laboratory ELISA to DFA with calculated sensitivity and specificity

Reference laboratory test	Sensitivity (95% CI)	Specificity (95% CI)	Prevalence adjusted % agreement^a^
Ova and Parasite (O&P) Test	81.2 (72.4–87.7)	93.4 (85.2–97.5)	92.7
ProSpecT Microtiter Plate ELISA	94.1 (87.4–97.5)	97.4 (90.4–99.8)	97.2

^a^Percent agreement was adjusted for reported prevalence of infection determined by DFA as described in materials and methods

### In-clinic test results

Among the in-clinic test results, there was a single invalid result with Abaxis VetScan (positive control line did not appear) and therefore there were only 176 comparisons with this test. In all other cases, there were 177 valid results. The in-clinic tests results were compared to results of DFA (Table 2). Sensitivity ranged from 70.0% for the VetScan test to 87.1% for the SNAP test, specificity ranged from 71.0% for the Witness test to 93.4% for the SNAP test and prevalence adjusted agreement ranged from 71.2% for the Witness test to 93.0% for the SNAP test. In-clinic test results were also compared to results of the microtiter plate format ELISA, values for positive and negative agreement and overall agreement are shown in Table 3. Among the in-clinic rapid tests, SNAP *Giardia* showed the highest agreement with DFA and the microtiter plate format ELISA.

**Table 2:** Statistical results of four in-clinic antigen tests for *Giardia* using DFA as the reference standard with calculated sensitivity and specificity

In-clinic test	Sensitivity (95% CI)	Specificity (95% CI)	Prevalence adjusted % agreement^a^
SNAP *Giardia*	87.1 (79.1–92.5)	93.4 (85.2–97.5)	93.1
Anigen Rapid CPV-CCV-*Giardia* Antigen Test	80.2 (71.3–86.9)	80.3 (69.8–87.8)	80.3
Witness *Giardia* Test	73.3 (63.9–81.0)	71.1 (60.0–80.1)	71.2
VetScan Canine *Giardia* Rapid Test	70.00 (60.4–78.1)	85.5 (75.7–91.9)	84.7
Ova and Parasite Test	81.2 (72.4–87.7)	93.4 (85.2–97.5)	92.7

^a^Percent agreement was adjusted for reported prevalence of infection determined by DFA as described in materials and methods

**Table 3:** Statistical results of four in-clinic antigen tests for *Giardia* using ProSpecT ELISA *Giardia* Test as the reference standard with calculated positive and negative percent agreement

In-clinic test	% Positive agreement (95% CI)	% Negative agreement (95% CI)	Prevalence adjusted % agreement^a^
SNAP *Giardia*	91.8 (84.4–96.0)	95.0 (87.5–98.4)	94.8
Anigen Rapid CPV-CCV-*Giardia* Antigen Test	80.4 (71.3–87.2)	77.5 (67.1–85.4)	77.7
Witness *Giardia* Test	77.3 (68.0–84.6)	73.8 (63.1–82.2)	74.0
VetScan Canine *Giardia* Rapid Test	72.9 (63.2–80.8)	86.3 (76.9–92.3)	85.6

^a^Percent agreement was adjusted for reported prevalence of infection determined by DFA as described in materials and methods

## Discussion

We sought to compare the performance of four commercially available in-clinic assays designed to detect *Giardia* infection in dogs. Although we used the O&P test to select samples to ensure a sufficient population of negative and positives, we used direct immunofluorescence as the gold standard because several studies have demonstrated its accurate performance compared to traditional flotation methods. We chose to include the microtiter plate format test for comparison because the test detects soluble *Giardia* specific cell wall antigen shed in the local environment and is used for high throughput screening in reference laboratories. In comparing the microtiter plate format ELISA results to the DFA results, ELISA had a sensitivity of 94.1%, and a specificity of 97.4% (Table 1).

Results for the comparison of the in-clinic tests to the DFA and the microtiter plate format ELISA standard are shown in Tables 2 and 3, respectively. In all three measures of performance, SNAP had the highest concordance with the DFA and the ELISA standards among the tests.

We also used DFA results to retrospectively evaluate performance characteristics of the O&P method (Table 1) and compared these to those found for the 4 in-clinic tests compared to DFA shown in Table 2. We observed that the performance of SNAP *Giardia* was in close agreement with the O&P method while each of the 3 remaining in-clinic tests showed reduced values for percent positive agreement, percent negative agreement and prevalence adjusted agreement relative to this method.

The performance of SNAP *Giardia* has been studied by several groups [6, 10, 11]. To our knowledge, this is the first study to evaluate multiple tests currently available for detection of *Giardia* including several new rapid in-clinic tests. We found the performance of rapid in-clinic tests varied by comparing to different reference standards. There could be a myriad of factors attributable to the observed variability. The SNAP *Giardia* is an ELISA-based assay designed for in-clinic use and the methodology has been described in detail in a recent technical review [13]. In the same journal issue dealing with “Point of Care Tests in Veterinary Medicine”, the lateral flow methodology, which is the basis for the other three in-clinic tests, is also described in detail [14]. Many specific components of each individual test are proprietary. It is not clear what specific differences in the in-clinic assayed may have caused the observed performance differences between the tests.

## Conclusions

We found that results of rapid in-clinic *Giardia* assays can vary amongst tests. Overall, of the in-clinic tests evaluated, the SNAP test most closely mirrored the results of the DFA and the microtiter plate ELISA and was the only test with a prevalence adjusted agreement greater than the O&P method. The major conclusion of this study is that among the in-clinic tests evaluated, *Giardia* SNAP is the most reliable method for detection of *Giardia* in canine stool samples.

## Abbreviations

DFA: Direct immunofluorescence assay
ELISA: Enzyme-linked immunosorbent assay
IRL: IDEXX Reference Laboratories
O&P: Ova and parasite testing, zinc sulfate fecal flotation
PBS: Phosphate-buffered saline 0.02M pH 7.4
ZnSO_4_: Zinc sulfate flotation solution
